# Acupuncture for persistent atrial fibrillation after catheter ablation: study protocol for a pilot randomized controlled trial

**DOI:** 10.1186/s13063-020-04967-y

**Published:** 2021-01-07

**Authors:** Ying Lin, Xian Wang, Xue-Bin Li, Bang-Qi Wu, Zhao-Hui Zhang, Wei-Hua Guo, Cun-Cao Wu, Xin Chen, Ming-Long Chen, Zhong Dai, Fu-Yan Chen, Rui Zhu, Chu-Xi Liang, Yun-Peng Tian, Gang Yang, Chao-Qun Yan, Jing Lu, Hai-Ying Wang, Jin-Ling Li, Jian-Feng Tu, He-Wen Li, Dan-Dan Yang, Fang-Ting Yu, Yu Wang, Jing-Wen Yang, Guang-Xia Shi, Shi-Yan Yan, Li-Qiong Wang, Cun-Zhi Liu

**Affiliations:** 1 Acupuncture Research Center, School of Acupuncture-Moxibustion and Tuina, Beijing University of Chinese Medicine, No. 11, Bei San Huan Dong Lu, Chaoyang District, Beijing, 100029 China; 2 Dongzhimen Hospital Affiliated to Beijing University of Chinese Medicine, Beijing University Cardiology Research Institute of Traditional Chinese Medicine, Beijing, 100700 China; 3 Department of Cardiology, Peking University People’s Hospital, Beijing, 100044 China; 4 National Clinical Research Center for Chinese Medicine Acupuncture and Moxibustion, First Teaching Hospital, Tianjin University of Traditional Chinese Medicine, Tianjin, 300193 China; 5 Department of Acupuncture and Moxibustion, The First Affiliated Hospital of Nanjing Medical University, Nanjing, 210029 China; 6 Department of Cardiology, Dongzhimen Hospital Affiliated to Beijing University of Chinese Medicine, Beijing, 100700 China; 7 Department of Cardiology, Tianjin First Center Hospital, Tianjin, 300192 China; 8 Department of Cardiology, The First Affiliated Hospital of Nanjing Medical University, Nanjing, 210029 China; 9 Department of Traditional Chinese Medicine, Peking University People’s Hospital, Beijing, 100044 China; 10 Department of Acupuncture and Moxibustion, Dongzhimen Hospital Affiliated to Beijing University of Chinese Medicine, Beijing, 100700 China; 11 Department of Acupuncture and Moxibustion, Beijing Hospital of Traditional Chinese Medicine Affiliated to Capital Medical University, Beijing, 100010 China

**Keywords:** Acupuncture, Atrial fibrillation burden, Catheter ablation, Persistent atrial fibrillation, Randomized controlled trial

## Abstract

**Background:**

Atrial fibrillation (AF) is a common arrhythmia, which is closely related to cardiovascular morbidity and mortality. Although acupuncture is used in the treatment of AF, the evidence is insufficient. The objective of this pilot trial is to evaluate the feasibility, preliminary efficacy, and safety of acupuncture in reducing AF burden for persistent AF after catheter ablation (CA).

**Methods and design:**

This will be a multi-center, 3-arm, pilot randomized controlled trial in China. Sixty patients in total will be randomly assigned to the specific acupoints group, the non-specific acupoints group, or the non-acupoints group in a 1:1:1 ratio. The whole study period is 6 months, including a 3-month treatment period and a 3-month follow-up period. All patients will receive 18 sessions of acupuncture over 12 weeks after CA and appropriate post-ablation routine treatment. The primary outcome is AF burden at 6 months after CA measured by electrocardiography patch that can carry out a 7-day continuous ambulatory electrocardiographic monitoring. The secondary outcomes include AF burden at 3  months after CA, recurrence of AF, quality of life, etc. The adverse events will also be recorded.

**Discussion:**

This pilot study will contribute to evaluating the feasibility, preliminary efficacy, and safety of acupuncture in reducing AF burden for persistent AF after CA. The results will be used for the sample size calculation of a subsequent large-scale trial.

**Trial registration:**

Chinese Clinical Trial Registry ChiCTR2000030576. Registered on 7 March 2020.

**Supplementary information:**

**Supplementary information** accompanies this paper at 10.1186/s13063-020-04967-y.

## Background

Atrial fibrillation (AF), as a common arrhythmia, increases in prevalence with advancing age [1]. Patients with AF have a 4–5 times greater risk of ischemic stroke than non-AF patients [2], resulting in 20% mortality and 60% disability when a stroke occurs [3]. AF triples the risk of heart failure [4] and doubles the risk of myocardial infarction [5]. In addition, AF is associated with a decreased quality of life and depression [6].

Catheter ablation (CA) is an invasive procedure used to treat AF that uses radiofrequency or other energy sources to isolate portions of the left atrium, such as the pulmonary veins, and other areas. For paroxysmal AF, although outcomes have improved over the last 20 years, success rates of CA, defined as freedom from recurrent AF, have plateaued in the 60–70% range [7]. For persistent AF, success rates are even more unfavorable at 45 to 60% at 1 year [8, 9]. There remain unmet needs in improving the ability to restore and sustain sinus rhythm [6]. Acupuncture, as a nonpharmacologic therapy, has been widely applied in cardiovascular diseases [10–12]. Acupuncture plays a role in regulating the autonomic nervous system in the treatment of many diseases [13–15]. The prevention effect of acupuncture on the recurrence of AF may have been achieved in this way. Previous trials have indicated that acupuncture could prevent persistent AF recurrence after electrical cardioversion or CA [16, 17]. However, in these trials, only the recurrence rate was evaluated, and there was no quantitative index like AF burden (the percentage of time in AF during a monitoring period) [18] and quality of life assessment was overlooked. Through a relatively simple detection method, the recurrence rate may be underestimated. Both are single-center studies, which could result in bias. As for the control setting, the blinding method cannot be completely achieved and the accurate efficacy of acupuncture was unable to be measured in the studies. Therefore, it is difficult to draw sound conclusions from the results of these trials due to the existing limitations of their design. In this context, we designed a multi-center, placebo-controlled pilot randomized trial to evaluate the feasibility, preliminary efficacy, and safety of acupuncture. AF burden will be the primary outcome and the recurrence rate will be measured by a relatively superior detection method.

It is well known that the efficacy of acupuncture is related to the acupoints selected. Some acupoints are called specific acupoints in traditional Chinese medicine (TCM), which have special properties, therapeutic effects, and a specific name [19, 20]. Specific acupoints have been applied in the treatment of many diseases [10, 21, 22], which are believed to be a group of acupoints superior to others in treating specific diseases. The aim of our study is as follows: (i) to explore the feasibility and provide power calculations for a future large-scale clinical trial, (ii) to explore the efficacy and safety of acupuncture as adjunctive therapy of routine care in reducing AF burden among patients with persistent AF after CA, and (iii) to examine whether the specific acupoints are superior to either non-specific acupoints or non-acupoints in therapeutic efficacy.

## Methods/design

### Study design

**Fig. 1:**
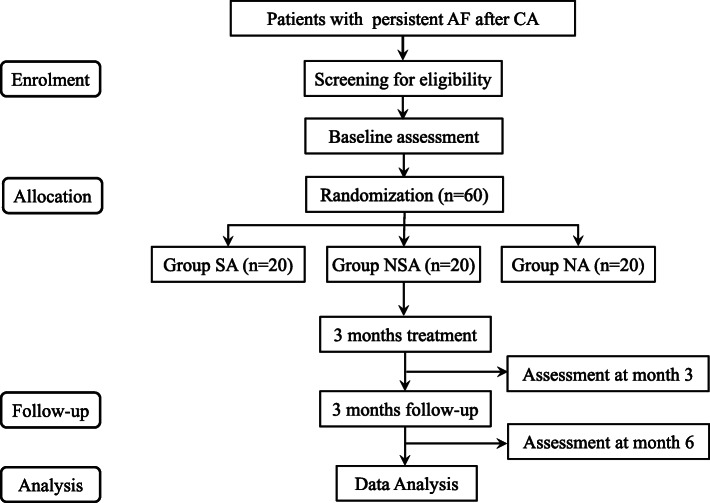
Flowchart of trial procedures

This multi-center, 3-arm, assessor and statistician blinded, pilot randomized controlled trial (RCT) will be conducted at four centers in China: Dongzhimen Hospital Affiliated to Beijing University of Chinese Medicine; Peking University People’s Hospital; The First Affiliated Hospital of Nanjing Medical University; Tianjin University of Traditional Chinese Medicine. Eligible patients will be enrolled from inpatient units of the cardiology departments at these hospitals. The whole study period will be 6 months and consist of a 3-month treatment period and a 3-month follow-up period. The CONSORT statement [23] and STRICTA guidelines [24] for acupuncture studies have been used as a framework for the methodology in this study. Besides, this protocol is reported following the SPIRIT guidelines (Additional file 1) [25]. The flowchart of this study is shown in Fig. 1.

### Participants

Patients with persistent AF will be diagnosed according to the 2016 ESC guidelines [6]. Persistent AF is defined as AF that lasts for more than 7 days, including episodes that are terminated by cardioversion after 7 days or more [6].

#### Inclusion criteria

1. Persistent AF lasting no more than 3 years.
2. Age between 18 and 75 years old (male or female).
3. No acupuncture treatment within the previous 6 months.

#### Exclusion criteria

1. Severe heart failure (New York Heart Association class III or IV).
2. Echocardiographic parameters: left ventricular ejection fraction < 40%, left atrium diameter > 5.0 cm.
3. Severe lung, liver, kidney disease, or other serious primary diseases.
4. Skin allergy to electrode patches for electrocardiographic monitoring.
5. The estimated life expectancy of < 1 year.

### Ablation procedure

Physicians who perform ablations are required to have done 100 cases prior to this trial. All centers will cooperate with a relatively uniform standard of ablation procedures. All antiarrhythmic drugs (AADs), except amiodarone, will be discontinued for at least five half-lives before the procedure. Amiodarone will be recommended to be discontinued 3 months before the ablation procedure. Warfarin or novel oral anticoagulants should be taken for at least 3 weeks before CA. Transesophageal echocardiography will be performed on the day of CA or 1 day before CA to rule out left atrial thrombus.

All patients will undergo pulmonary vein isolation (PVI) by radiofrequency ablation. Selection of the type of ablation catheter, settings of power and irrigation, and the use of 3-dimensional mapping systems will be at the discretion of the physicians performing CA. It is regarded as an endpoint that the complete entrance and exit block of all pulmonary vein antra are confirmed in PVI. If the patient is still in a state of AF after PVI, electrical cardioversion will be performed. Patients who fail to be converted to sinus rhythm after PVI or electrical cardioversion will be excluded from this study.

### Randomization and blinding

All the patients will be randomly assigned (in a 1:1:1 ratio) to the specific acupoints (SA) group, non-specific acupoints (NSA) group, or non-acupoints (NA) group. NA group is the control group in this trial. Stratification will be performed by center and the method of block randomization will be applied in each center. An independent statistician who is not involved in treatment and outcome assessment will be in charge of computer-generated randomization sequence using SAS 9.3 software (SAS Institute, Cary, NC, USA). The allocation schedule will be carried out by an independent researcher who will be responsible for providing random numbers and group assignments to recruiters at each center through a centralized telephone randomization procedure. The patients, outcome assessors, and statisticians will be blinded. However, the acupuncturists will not be blinded due to the nature of acupuncture. The allocations will not be revealed until the data’s statistical analysis is completed.

### Interventions

The acupuncture regimens were determined according to the clinical practice of acupuncture experts and literature on acupuncture treatment of AF [16, 17, 26, 27]. All acupuncture operations will be performed by professional acupuncturists who have obtained a Chinese medicine practitioner license with more than 3 years of clinical experience. Besides, all patients will receive relatively uniform post-ablation management after CA.

**Fig. 2:**
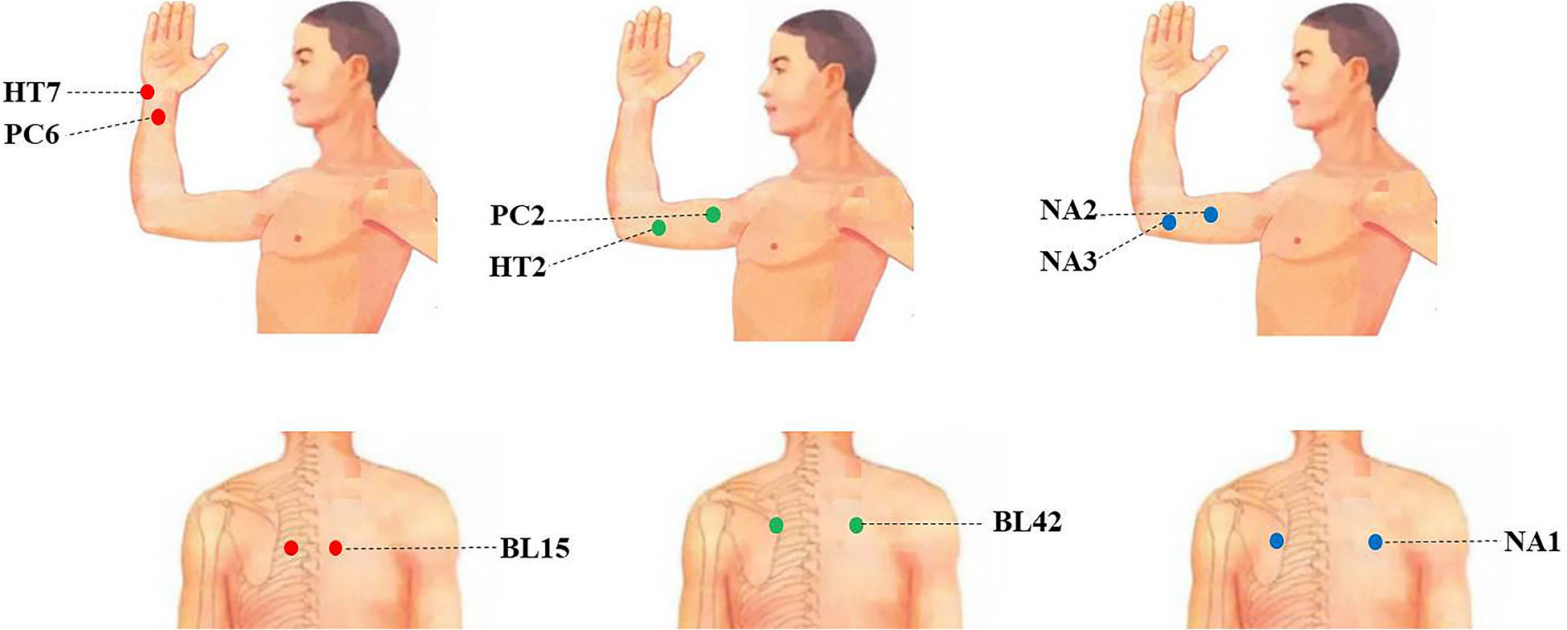
Locations of acupoints and non-acupoints

Before the study, all acupuncturists will be specially trained in the standardized operation procedure of acupuncture, including accurate positioning of acupoints, operational methods, and so on. Besides, brochures showing the information on the standardized operation in detail will be provided to the acupuncturists for reference. Within 3 months after CA (blanking period), patients will receive acupuncture treatment twice per week for weeks 1–6 and once per week for weeks 7–12 (18 sessions in total). Sterilized disposable steel needles (Hwato disposable acupuncture needle; Suzhou, Jiangsu, China) will be used in the treatment and in sizes of 0.30 mm × 25 mm or 0.30 mm × 40 mm. Standardized prescriptions will be used in the three groups. Bilateral *xinshu* (BL15), *neiguan* (PC6), and *shenmen* (HT7) will be selected in the SA group. Bilateral pohu (BL42), tianquan (PC2), and qingling (HT2) will be selected in the NSA group. The code and location of the acupoints are consistent with the WHO standards [28]. The exact locations of all acupoints are listed in Table 1 and marked in Fig. 2. The three non-acupoints chosen for the NA group are separated from known acupoints. The locations of non-acupoints are shown in Table 2 and also marked in Fig. 2.

**Table 1:** Locations of acupoints for specific acupoints group and non-specific acupoints group

	Acupoint	Location
Specific acupoints	Xinshu (BL15)	In the upper back region, at the same level as the inferior border of the spinous process of the fifth thoracic vertebra (T5), 1.5 cun^a^ lateral to the posterior median line.
	Neiguan (PC6)	On the anterior aspect of the forearm, between the tendons of the palmaris longus and the flexor carpi radialis, 2 cun proximal to the palmar wrist crease.
	Shenmen (HT7)	On the anteromedial aspect of the wrist, radial to the flexor carpi ulnaris tendon, on the palmar wrist crease.
Non-specific acupoints	Pohu (BL42)	In the upper back region, at the same level as the inferior border of the spinous process of the third thoracic vertebra (T3), 3 cun lateral to the posterior median line.
	Tianquan (PC2)	On the anterior aspect of the arm, between the long head and short head of the biceps brachii muscle, 2 cun distal to the anterior axillary fold.
	Qingling (HT2)	On the medial aspect of the arm, just medial to the biceps brachii muscle, 3 cun superior to the cubital crease.

^a^1 cun (≈ 20 mm) is defined as the width of the interphalangeal joint of the patient’s thumb

**Table 2:** Locations of non-acupoints group

Non-acupoints	Location
NA1	In the middle of Shentang (BL44) and Tianzong (SI11) points
NA2	In the middle of Tianquan (PC2) and Qingling (HT2) points
NA3	In the middle of Quze (PC3) and Qingling (HT2) points

#### SA group

All four acupoints in this group are specific acupoints. Acupuncture is performed in a certain sequence of acupoints: BL15, PC6, and HT7. Firstly, BL15 will be punctured obliquely to a depth of 1.0–1.6 cm. Then, the needles will be stimulated (lift and thrust or twirl and rotate the needles) manually to achieve the Deqi sensation (the sensation including soreness, numbness, distention, or heaviness accompanied by acupuncture). The stimulation will continue for 1 min, and then the needles will be pulled out. Secondly, PC6 will be punctured perpendicularly 1.0–2.0 cm. HT7 will be punctured perpendicularly 0.6–1.0 cm. PC6 and HT7 are also stimulated to achieve Deqi. After that, needles will be retained in place for 30 min. 

#### NSA group

Non-specific acupoints are a group of acupoints that are not thought to have similar specific effects to specific acupoints. The nearby non-specific points belonging to the same meridians of the specific acupoints group for treatment were selected as in some previous studies [21, 22]. The sequence of acupuncture will be BL42, PC2, and HT2. BL42 will be punctured obliquely 1.0–1.6 cm using identical operation with BL15. PC2 will be punctured perpendicularly 2.0–3.0 cm. HT2 will be punctured perpendicularly 1.0–2.0 cm. The acupuncturist will use the same operation as PC6 and HT7 at PC2 and HT2 in the SA group.

#### NA group

Shallow puncture (2–3 mm in depth) will be performed without obtaining Deqi. It will be safe and not do any harm to the patients. Acupuncture will follow this sequence: NA1, NA2, NA3. Needles in NA2 and NA3 should be left for 30 min, whereas needles will not be retained in NA1.

#### Post-ablation management

Electrical cardioversion is allowed within 3 months after CA (blanking period). The blanking period refers to a period of 3 months after any form of cardiac ablation during which arrhythmias are not considered. Patients who experience AF recurrence after the blanking period will be allowed to start or resume AADs or undergo a repeat ablation. According to the guidelines of the ESC [6] and Chinese guidelines [29] for the management of AF, all patients will receive routine medication after CA. The drugs protocol includes anticoagulation (at least 8 weeks after CA), AADs (continued 3 months after CA), and proton pump inhibitor (4 weeks). Moreover, patients are required to keep a detailed record of their medication use, including name, duration, and dose.

### Outcomes

#### Primary outcome measurement

The primary outcome is the AF burden at 6 months after CA. AF burden will be measured by NS-SP-B-01 smart patch (Ensense Biomedical Technologies Co., Ltd., Shanghai, China). The smart patch will carry out continuous ambulatory electrocardiographic monitoring for 7 days. AF burden is expressed in the form of a percentage, which specifically refers to the proportion of time in AF within 7-days’ long for one patient in this study (range 0–100%). When the monitoring is complete, the patient will return the smart patch and the researchers can get the recordings. After the researcher transmits the recordings to the Ensense Company, the recordings will be analyzed by an appropriate algorithm. The results then will be reviewed by certified cardiac technicians to assure analysis quality and generate a report transmitted to the researchers.

#### Secondary outcome measurements

##### AF burden at 3 months after CA

AF burden at 3 months after CA will be measured by the same method as the primary outcome. This outcome is to evaluate whether acupuncture can reduce the duration of AF after treatment.

##### Time to AF recurrence

Recurrence of AF within 6 months after CA will be measured, and it is defined as any atrial tachyarrhythmia including AF/ atrial flutter/atrial tachycardia lasting 30 s or longer after the 3-month blanking period. Patients will be instructed to obtain 12-lead electrocardiograph (ECG) or 24 h Holter in order to verify the recurrence whenever they experience AF-related symptoms (palpitations, dyspnea, chest pain, etc.). Additionally, recurrence will be monitored via the smart patch regardless of the existence of symptoms at the end of 3 and 6 months. All researchers are required to receive training in the procedure of electrocardiographic monitoring and the use of the NS-SP-B-01 smart patch. They will then be responsible for explaining the use of the device to the patients.

##### Atrial Fibrillation Effect and Quality of Life Scale (AFEQT)

The AFEQT [30] is a questionnaire specially designed to assess the quality of life in atrial fibrillation patients. The assessment will take place at baseline and 3 and 6 months. A summary score and subscale scores in the following 3 domains: symptoms, daily activities, and treatment will be calculated. Summary and subscale scores range from 0 to 100 (complete to no AF-related disability).

##### Heart rate variability

Heart rate variability will be generated while monitoring AF burden with the smart patch to quantitatively evaluate the balance between the sympathetic and vagus nerves at 3 and 6 months.

##### Credibility and expectancy

A Credibility/Expectancy Questionnaire [31] will be used to measure the patients’ attitudes toward acupuncture before treatment.

##### Blinding assessment

Blind evaluation will be conducted in order to evaluate whether the blind method is successful. After treatments in weeks 6 and 12, all patients will be asked to guess whether the needles go deep or shallow layers in the acupuncture treatment they receive. Before the treatment, one or two doctors of each center will be in charge of recruiting the patients and gaining informed consent from them. The patients will be informed: “There are three different groups of acupuncture with three different acupoint selection schemes. You are equally likely to be assigned to all three groups, and previous studies have shown that all three methods were associated with positive outcomes in clinical studies.”

##### Others

They include the number of electrical cardioversion within the blanking period, the number of repeat CA after the blanking period, and the number of AF-related hospitalizations.

### Adverse events

Any adverse events and how they are dealt with will be recorded from baseline to 6 months. These adverse events specifically included bleeding, hematoma, fainting, severe pain, and local infection. If any serious adverse event occurs to the patient, which will be immediately reported to the primary investigator and all details will be recorded. The patient will be withdrawn from the trial.

### Data management

**Fig. 3:**
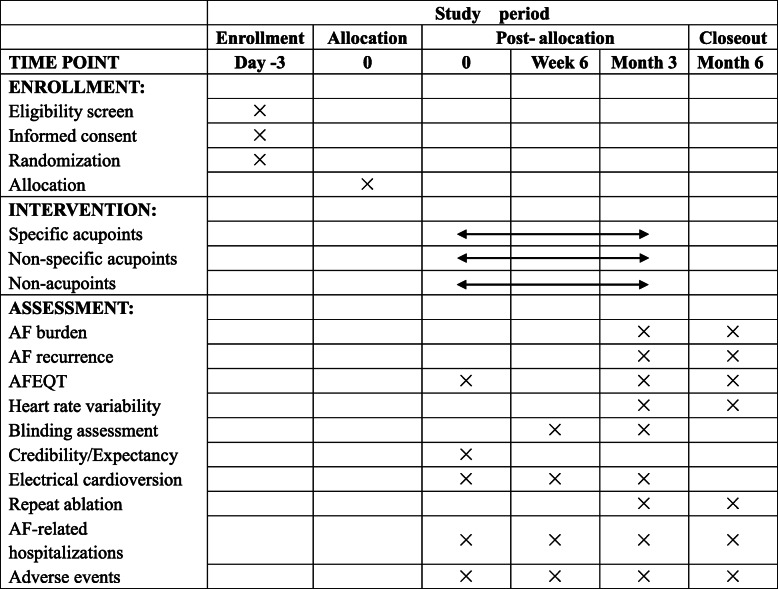
Schedule of enrollment, intervention, and assessments (SPIRIT figure). AF, atrial fibrillation; AFEQT, Atrial Fibrillation Effect on Quality of Life

Detailed time points of outcome assessments are provided in Fig. 3. Throughout this study, the data of all patients will be recorded on the case report form (CRF). When the forms are completed, the data will be entered into the Excel spreadsheet independently by two researchers. There will be a data manager to compare the two sets of data. If any differences are found, they will be corrected according to the original CRFs. All documents related to the research, including the paper files and electronic documents, will be well preserved for no less than 5 years after publication. The first author or corresponding author will provide the original data if anyone puts forward questions about the published data. It will be protected and never be disclosed that the patients’ personal privacy information includes name, gender, age, address, and telephone number. The ethics committee of Beijing University of Chinese medicine will audit trial conduct every 12 months.

### Sample size

The purpose of this pilot study is to evaluate the efficacy and safety of acupuncture in the treatment of AF and the feasibility for a further large-scale clinical trial. We did not find a suitable reference to calculate the sample size, but a minimum sample size of the clinical trial with 20 patients per group is used instead [32]. A total of 60 patients will be randomly assigned equally to SA, NSA, or NA group. This trial will provide data for sample size calculations of a future large-scale RCT.

### Statistical analysis

Baseline characteristics will be summarized by the treatment group. Continuous variables will be described as mean ± standard deviations (normal distribution), or medians and interquartile ranges (non-normal distributions). Analysis of variance (ANOVA) or Kruskal-Wallis test (if normality is violated) will be applied in the comparison among three groups. Categorical variables will be described as frequency (percentage) and compared by *χ*^2^ test or Fisher’s exact test etc. As for the primary outcome, AF burden is expressed in the form of a percentage, which specifically refers to the proportion of time in AF within 7-days’ long for one patient in this study (range 0–100%). The null hypothesis that AF burden would be equivalent among the three arms (H0: effect of SA =effect of NSA = effect of NA) will be tested. The means of AF burden at 6 months of the three arms will be compared using ANOVA based on the preliminary assessment of the data distributions. If the ANOVA test among 3 arms is significant, the pairwise comparison will be performed by the least significant difference (LSD) test, and mean differences of pairwise comparison will be reported as effect measure. If AF burden is a skewed distribution, the data will be transformed to a normal distribution by corresponding methods for further comparison, and the same statistical analysis strategy will be adopted. The comparisons of AF recurrence between the three arms at 6 months will be performed based on a time-to-first-event analysis using the log-rank test. Kaplan-Meier cumulative AF recurrence rates will be calculated for each arm, with event or censoring times measured from the time of randomization. Hazard ratios (HRs) with associated 95% CIs will be derived using the Cox proportional hazards model.

All efficacy analyses will be based on the intention-to-treat principle. Missing data on the primary outcomes will be imputed by multiple imputations. An independent statistician who is blinded will conduct the statistical analysis using SAS 9.3. We will set a significance level of *P* < 0.05 (two-sided) without adjustment on account of the values considered explorative.

### Ethics and dissemination

The trial will be conducted in accordance with the Declaration of Helsinki and has been approved by the Ethics Committee of Beijing University of Chinese Medicine (No. 2020BZHYLL0106). Patients will be included only after the details of the study explained to them and signing informed consent forms. The results of the trial will be published in a peer-reviewed academic journal.

## Discussion

AF is one of the leading causes of stroke and cardiovascular diseases. It is of great significance to find combination therapy to maintain sinus rhythm. This pilot study is designed to evaluate the feasibility, efficacy, and safety of acupuncture in reducing AF burden in a specific group of patients with persistent AF after CA.

Previously, an RCT was conducted to prevent AF recurrence after electrical cardioversion with acupuncture in persistent AF [16]. AF was detected only by 12-lead ECG, which could underestimate the recurrence rate of AF. Another study was designed to determine whether acupuncture could reduce the early recurrences of AF after CA in persistent AF [17]. In the study, the measured recurrence rate of AF was the early recurrence rate within the blanking period, and the long-term prognosis was not studied, which is a more appropriate endpoint. In addition, neither of the two studies evaluated the quality of life that is of great concern in AF patients [33, 34].

The recurrence rate is often used as the primary outcome in clinical trials on AF. However, recurrence is a binary concept, reflecting only the presence and absence. In contrast, AF burden is the primary outcome of our study, which can represent the quantity or amount of AF that one person has. It helps analyze the therapeutic efficacy quantitatively. The AF recurrence after the blanking period will be set as a significant secondary outcome in our study. We will both use 12-lead ECG and 24-h Holter during the monitoring, which may ensure AF recurrence detected in time. Moreover, we will evaluate the quality of life through AFEQT. The control group is the NA group in which shallow insertion at non-acupoint is a kind of sham treatment of acupuncture. Shallow insertion in non-acupoints applied as a sham control was established in the light of two literature reviews [35, 36]. In addition, this placebo control has been verified to be successful in blinding Chinese patients suffering from postprandial distress syndrome [37] and chronic severe functional constipation [38]. Compared with a blank control group, sham acupuncture can largely eliminate the interference of non-specific effects. We will choose the acupoints selection scheme with better efficacy through the comparison between group SA and group NSA, which will be applied in the future large-scale RCT.

There are some limitations to our study. First, we will not be able to monitor AF at all the times by using implant loop recorder, since very few patients are willing to do invasive monitoring. Second, as a pilot study, the sample size is small, and it is impossible to evaluate the long-term efficacy without insufficient follow-up time in this study. Nevertheless, after that, we will conduct a large-scale RCT to solve these problems. Third, acupuncturists cannot be blinded during acupuncture owing to the nature of the intervention, which may generate bias.

## Trial status

This trial is currently recruiting patients.

## Supplementary Information

**Additional file 1.** Completed Standard Protocol Items: Recommendation for Interventional Trials (SPIRIT) 2013 Checklist: items addressed in this clinical trial protocol.

**Additional file 2.** STRICTA 2010 checklist of information to include when reporting interventions in a clinical trial of acupuncture (Expansion of Item 5 from CONSORT 2010 checklist).

## Data Availability

All individual participants’ data collected during the trial will be available for anyone who wishes to access the data immediately following publication in accordance with FAIR principles.

## Abbreviations

AF: Atrial fibrillation
CA: Catheter ablation
TCM: Traditional Chinese medicine
RCT: Randomized controlled trial
PVI: Pulmonary vein isolation
AADs: Antiarrhythmic drugs
SA: Specific acupoints
NSA: Non-specific acupoints
NA: Non-acupoints
ECG: Electrocardiograph
AFEQT: Atrial Fibrillation Effect and Quality of Life Scale
CRF: Case report form
ANOVA: Analysis of variance
LSD: Least significant difference
HRs: Hazard ratios
