# Supplementary material for: Acupuncture for persistent atrial fibrillation after catheter ablation: study protocol for a pilot randomized controlled trial
Source: Trials. 2021 Jan 7;22:35. doi: 10.1186/s13063-020-04967-y (PMC7792186; doi:10.1186/s13063-020-04967-y)
Supplement: Supplementary file 2 — Additional file 2. STRICTA 2010 checklist of information to include when reporting interventions in a clinical trial of acupuncture (Expansion of Item 5 from CONSORT 2010 checklist). [file 13063_2020_4967_MOESM2_ESM.doc]

***Citation:*** *MacPherson H, Altman DG, Hammerschlag R, Youping L, Taixiang W, White A, Moher D; STRICTA Revision Group. Revised STandards for Reporting Interventions in Clinical Trials of Acupuncture (STRICTA): extending the CONSORT statement. PLoS Med. 2010 Jun 8;7(6):e1000261*

**Table 1: STRICTA 2010 checklist of information to include when reporting interventions in a clinical trial of acupuncture (Expansion of Item 5 from CONSORT 2010 checklist)**

| **Item** | **Detail** | **Page No** |
| --- | --- | --- |
| **1. Acupuncture rationale** | 1a) Style of acupuncture (e.g. Traditional Chinese Medicine, Japanese, Korean, Western medical, Five Element, ear acupuncture, etc) | 7 |
|  | 1b) Reasoning for treatment provided, based on historical context, literature sources, and/or consensus methods, with references where appropriate | 7,10 |
|  | 1c) Extent to which treatment was varied | 10-12 |
| **2. Details of needling** | 2a) Number of needle insertions per subject per session (mean and range where relevant) | 11,12 |
|  | 2b) Names (or location if no standard name) of points used (uni/bilateral) | 11,12 and Fig. 2,Fig. 3,Table 1,Table2 |
|  | 2c) Depth of insertion, based on a specified unit of measurement, or on a particular tissue level | 11,12 |
|  | 2d) Response sought (e.g. *de qi* or muscle twitch response) | 11,12 |
|  | 2e) Needle stimulation (e.g. manual, electrical) | 11,12 |
|  | 2f) Needle retention time | 11,12 |
|  | 2g) Needle type (diameter, length, and manufacturer or material) | 11 |
| **3. Treatment regimen** | 3a) Number of treatment sessions | 11 |
|  | 3b) Frequency and duration of treatment sessions | 11 |
| **4. Other components of treatment** | 4a) Details of other interventions administered to the acupuncture group (e.g. moxibustion, cupping, herbs, exercises, lifestyle advice) | 13 |
|  | 4b) Setting and context of treatment, including instructions to practitioners, and information and explanations to patients | 8,10,11,15 |
| **5. Practitioner background** | 5) Description of participating acupuncturists (qualification or professional affiliation, years in acupuncture practice, other relevant experience) | 10 |
| **6. Control or comparator interventions** | 6a) Rationale for the control or comparator in the context of the research question, with sources that justify this choice | 19 |
|  | 6b) Precise description of the control or comparator. If sham acupuncture or any other type of acupuncture-like control is used, provide details as for Items 1 to 3 above. | 12 |
